# Role of Grape-Extractable Polyphenols in the Generation of Strecker Aldehydes and in the Instability of Polyfunctional Mercaptans during Model Wine Oxidation

**DOI:** 10.1021/acs.jafc.1c05880

**Published:** 2021-12-13

**Authors:** Elena Bueno-Aventín, Ana Escudero, Purificación Fernández-Zurbano, Vicente Ferreira

**Affiliations:** † Laboratorio de Análisis del Aroma y Enología (LAAE), Departamento de Química Analítica, Universidad de Zaragoza, Instituto Agroalimentario de Aragón (IA2) (UNIZAR-CITA), C/Pedro Cerbuna 12, Zaragoza 50009, Spain; ‡ Instituto de Ciencias de la Vid y del Vino (Universidad de La Rioja, CSIC, Gobierno de La Rioja). Finca La Grajera, Logroño, La Rioja E-26007, Spain

**Keywords:** aroma, longevity, premox, shelf life, quinones, disulfides, nucleophiles, phenylacetaldehyde, methional, 3-mercaptohexanol

## Abstract

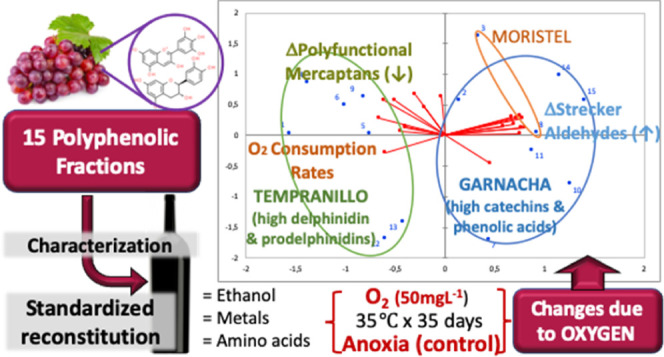

Polyphenolic fractions from Garnacha, Tempranillo, and Moristel grapes were reconstituted to form model wines of identical pH, ethanol, amino acid, metal, and varietal polyfunctional mercaptan (PFM) contents. Models were subjected to a forced oxidation procedure at 35 °C and to an equivalent treatment under strict anoxia. Polyphenolic profiles significantly determined oxygen consumption rates (5.6–13.6 mg L^–1^ day^–1^), Strecker aldehyde (SA) accumulation (ratios max/min around 2.5), and levels of PFMs remaining (ratio max/min between 1.93 and 4.53). By contrast, acetaldehyde accumulated in small amounts and homogeneously (11–15 mg L^–1^). Tempranillo samples, with highest delphinidin and prodelphinidins and smallest catechin, consume O_2_ faster but accumulate less SA and retain smallest amounts of PFMs under anoxic conditions. Overall, SA accumulation may be related to polyphenols, producing stable quinones. The ability to protect PFMs as disulfides may be negatively related to the increase in tannin activity, while pigmented tannins could be related to 4-methyl-4-mercaptopentanone decrease.

## Introduction

Wine longevity is a complex multifactor phenomenon in which the weight of the different factors is not well known. One of the key factors of wine longevity is related to its resistance to oxidation. This property can be defined as the ability of the wine, under an exposure to oxygen, to keep its color, avoid accumulation of acetaldehyde and Strecker aldehydes (SAs), and keep as long as possible labile varietal aroma compounds, such as polyfunctional mercaptans (PFMs).

The formation of acetaldehyde in the absence of free SO_2_ has been widely studied, although some details of the process are not completely understood. The hydrogen peroxide formed in the first two-electron reduction of O_2_, taken from an *o*-diphenol, reacts with Fe(II) cations to form the powerful hydroxyl radical, OH^•^. Once formed, this radical is a very powerful oxidant, which reacts at diffusion-controlled rates. It is, therefore, proposed that it reacts close to its site of production with the first potential substrate it encounters.^1^ This implies that most of it oxidizes ethanol to form 1-hydroxyethyl radical (1-HER), and this, in the presence of oxygen, forms 1-hydroxyethyl peroxyl which decomposes into acetaldehyde.^2,3^ However, the reaction is quite complex. It has been suggested that *o*-diphenols can quench the 1-HER radical, and it has been demonstrated that cinnamic acids are particularly efficient at trapping it.^4^ It has been also suggested that although the reaction of mercaptans with H_2_O_2_ is kinetically very slow (10^–2^ or 10^–3^ M^–1^ s^–1^ for cysteine),^5^ these compounds can reduce the 1-HER back to ethanol,^6^ which is kinetically much faster (10^9^ M^–1^ s^–1^).^7,8^ A recent report has shown that, quite paradoxically, some antioxidants such as ascorbic acid apparently inhibit the 1-HER radical but do not prevent the accumulation of acetaldehyde, suggesting that in fact this compound accelerates the oxidation of 1-HER into acetaldehyde.^9^ Finally, acetaldehyde could react with the nucleophilic positions of wine polyphenols, particularly in the A ring of the flavonoids, to form different combinations, such as ethylidene-bridged dimers or pyranoanthocyanins.^10^ Consequently, the accumulation of acetaldehyde in response to O_2_ consumption is very difficult to predict.

The SAs, isobutanal, 2-methylbutanal, isovaleraldehyde, methional, and phenylacetaldehyde, are powerful odor molecules, which, along with acetaldehyde, are mainly responsible for wine oxidative aroma.^11^ Different studies have demonstrated or suggested the existence of different SA formation routes. One of them is the own fermentation, in which these compounds can be formed via the Ehrlich pathway and remain unnoticed under the form of hydroxyalkylsulfonates, the nonvolatile adducts they form with SO_2_. These forms can regenerate free aldehydes during wine oxidation, as SO_2_ is consumed.^12^ The second and the most important formation pathway seems to be the Strecker degradation of the corresponding amino acids.^13^ This degradation requires an α-dicarbonyl, which can be a fermentation byproduct, such as methylglyoxal or diacetyl, or the quinones of *o*-diphenols formed during oxidation, for whose formation metal cations and oxygen are essential. Some authors have demonstrated that at high temperatures (80 and more than 130 °C), some polyphenols are more efficient than others for producing phenylacetaldehyde.^14,15^ Under those conditions, single nuclei *ortho*-diphenols, such as catechol, 4-methylcatechol, and 2,5-dihydroxybenzoic acid, or vicinal triphenols, such as pyrogallol or gallic acid, seem to be more efficient than flavanols, such as catechin or epicatechin (EC), in the accumulation of phenylacetaldehyde. The influence of polyphenols in the ability of wine to accumulate acetaldehyde and SAs has been indirectly suggested by partial least-squares (PLS) modeling. All models explaining the accumulation rates of aldehydes have in common negative coefficients for anthocyanins, which was therein interpreted as a consequence of their ability to quench aldehydes.^16^ Therefore, the ability of wine to accumulate SAs is related to the presence of the amino acid precursors, to its tendency to form amino acid reactive quinones, and to its capacity to quench formed aldehydes. Unfortunately, none of these three characteristics have been defined for the different wine polyphenols under wine-like conditions.

Regarding varietal aroma, the most oxygen-sensitive aroma compounds are PFMs, being the most important are 4-methyl-4-mercaptopentanone (4MMP), 3-mercaptohexanol (3MH), and its acetate, 3-mercaptohexyl acetate (MHA). These compounds are quite reactive. They can form disulfides as demonstrated by Roland et al.,^17^ but they can also react with wine quinones, as demonstrated by Nikolantonaki et al.^18,19^ Therefore, their stability will depend again on different compositional factors such as the wine ability to quench the 1-HER radical, the presence of other major mercaptans to form disulfides and the number and reactivity of quinones formed. It follows that such stability will be closely related to the wine polyphenolic composition but, again, the role of the different polyphenols is not known.

The main goal of the present research is to assess, specifically, the role played by the polyphenolic composition on the ability of wine models to accumulate SAs and to retain PFMs and other varietal aroma compounds during oxidation.

## Material and Methods

### Reagents and Standards

Hydrochloric acid (37%), sodium hydrogencarbonate, and sodium metabisulfite 97% were obtained from Panreac (Barcelona, Spain). l(+)-tartaric acid (99%), glycerol (99,5%), iron(II) chloride tetrahydrate (>99%), manganese(II) chloride tetrahydrate (>99%), copper(I) chloride (99,9%), l-leucine (Leu) (>98%), l-isoleucine (Ile) (>98%), d-valine (Val) (>98%), l-phenylalanine (Phe) (>98%), d-methionine (Met) (>98%), l-cysteine hydrochloride anhydrous (>98%), l-glutathione (GSH) reduced (>98%), hydrogen sulfide (≥99.5%), ethanethiol (97%), 2,4-dinitrophenylhydrazine (DNPH) (97%), and acetaldehyde (>99,5%) were obtained from Sigma-Aldrich Madrid, Spain, and malvidin 3-O-glucoside, ovalbumin (≥90%), (−)-EC (purity ≥90%), phloroglucinol, liquid chromatography (LC)–mass spectrometry (MS) grade formic acid used as the mobile phase additive, and all the solvents for the phloroglucinolysis reactions, extraction, isolation, and analysis were purchased from FLUKA Sigma-Aldrich St. Louis, USA. 4-Mercapto-4-methyl-2pentanone (4MMP) 1% in polyethylene glycol (PG) and 3-MHA were obtained from Oxford Chemicals (Hartlepool, U.K.). 3MH was obtained from Lancaster (Strasbourg, France), as 4-mercapto-4-methyl-2pentanone-d10 (4MMP-d10), 3-MHA-d5 (MHA-d5), and 3-mercaptohexanol-d5 (3MH-d5). LiChrolut EN sorbent, 1 mL cartridge and polytetrafluoroethylene frits, dichloromethane, and ethanol were purchased from Merck (Darmstadt, Germany). Sep Pak-C18 resins, prepacked in 10 g cartridges, were obtained from Waters (Ireland). l-Cysteine hydrochloride anhydrous (99%), sodium citrate trihydrate, and methanol of LC–MS LiChrosolv grade used for the preparation of mobile phases were obtained from Fluka. Sodium hydroxide 99%, high-performance LC (HPLC)-grade acetonitrile and *o*-phosphoric acid were purchased from Scharlab (Sentmenat, Spain). Isobutyraldehyde (Isobut) (99%), 2-methylbutanal (2MB) (95%), 3-methylbutanal (3MB) (95%), phenylacetaldehyde (PheAc) (95%) and methional (98%), 2-methylpentanal (98%), 3-methylpentanal (97%), and O-(2,3,4,5,6-pentafluorobenzyl)hydroxylamine hydrochloride (PFBHA) 98% were supplied by Merck USA. Phenylacetaldehyde-d2 (95%) and methional-d2 were purchased from Eptes (Vevey, Switzerland). Water was purified in a Milli-Q system from Millipore (Bedford, UK). Highest purity (>98%) grade(+)-catechin, (−)-EC, (−)-gallocatechin (GC), (−)-epigallocatechin (EGC), (−)-EC gallate (ECG), procyanidin B1, and procyanidin B2 were obtained from TransMIT PlantMetaChem (Gießen, Germany). The phloroglucinolated derivatives EC 4-phloroglucinol, EC-gallate 4-phloroglucinol, and EGC 4-phloroglucinol were prepared according to Arapitsas et al., 2021.^20^

### Polyphenolic and Aroma Fractions

The 15 polyphenolic aromatic fractions (PAFs) were extracted from 15 lots of grapes from three different Spanish wine-making regions (La Rioja, Ribera del Duero, and Somontano) and three different grape cultivars (7 from Tempranillo, 6 from Garnacha and 2 from Moristel), as described in Alegre et al.^21^ Briefly, 10 kg of grapes was collected at technological maturity, kept at 5 °C during the transport to the experimental cellar, destemmed and crushed in the presence of 50 mg/Kg of potassium metabisulfite and ethanol (adjusted to 15% v/v), and left in the dark at 13 °C for 7 days in closed recipients with no headspace after pressing to obtain the liquid mistella (ethanolic must), which after sterile filtration was stored at 5 °C in 750 mL wine bottles closed with a natural cork and no headspace. Then, 750 mL aliquots were dealcoholized by rotary evaporation at 23 °C (20 mbar) to a final volume of 410 mL and then extracted in a 10 g Sep Pak C18 cartridge. Sugars, acids, amino acids, and ions were removed by cleanup with water acidified at pH 3.5. PAFs were eluted with 100 mL of absolute ethanol and kept at −20 °C.

### Preparation of Model Wines

This operation was carefully carried out inside a glovebox (Jacomex) containing less that 1 ppm O_2_. The 100 mL ethanolic extracts were reconstituted with water containing 5 g/L tartaric acid, and pH adjusted at 3.5 and spiked with glycerol (5 g/L), FeCl_2_·4 H_2_O (5 mg/L), MnCl_2_·4 H_2_O (0,2 mg/L), and CuCl (0,2 mg/L) to form 750 mL of model wines 13.3% (v/v) in ethanol. The models were left to stand for 2 weeks within the anoxic chamber and were then spiked with 200 μg/L H_2_S, 25 μg/L ethanethiol, 10 mg/L cysteine, and 10 mg/L GSH and left under strict anoxia for 2 additional weeks. After this, the models were spiked with 10 mg/L of Leu, Ile, Val, Phe, and Met and with 100 μg/L of the three PFMs: 4MMP, MHA, and 3 MH. The anoxic controls were prepared by distributing three 60 mL aliquots of each model in three 60 mL screw-capped glass tubes (Wit Deluxe, Denmark), tightly closed and double vacuum bagged, including a layer of powder containing an O_2_ scavenger (AnaeroGen from Thermo Scientific Waltham, Massachusetts, United States) between both bags.

### Forced Oxidation Procedure

The model wines were taken out of the glovebox, saturated with air by vigorous shaking, and then distributed in 60 mL Wit-tubes of internal volume perfectly known and containing Pst3 Nomasense oxygen sensors to measure dissolved oxygen in the liquid sample. Each tube contained the volumes of liquid and headspace required to deliver 50 mg of O_2_ per L of liquid, as described by Marrufo-Curtido et al.^22^ Tubes were incubated in an orbital shaking thermostatic bath (Grant instruments OLS Aqua Pro) at 35 °C for 35 days. Dissolved oxygen was daily controlled.

### Chemical Characterization of the PAFs

The detailed analytical conditions are given in the Supporting Information. Anthocyanins were analyzed by ultra-HPLC–MS/MS, as described by Arapitsas et al.^23^ Flavanols, flavonols, and hydroxycinnamic acids were analyzed, as described by Vrhovsek et al.,^24^ by UHPLC–MS/MS. The mean degree of polymerization (mDP) was determined by UPLC–MS/MS analysis of the phloroglucinol reaction, as described by Arapitsas et al.^20^ Tannin activity and total and pigmented tannins were determined by UHPLC with photodiode array detection (280 and 520 nm) at four different temperatures (30, 35, 40, and 45 °C), as the specific enthalpy of interaction between tannins and a hydrophobic surface (polystyrene divinylbenzene HPLC column), as proposed by Yacco et al.^25^ The concentration of total and pigmented tannins were determined in the chromatogram made at 30 °C and they were reported in EC equivalents and area data, respectively.

### Chemical Characterization of Oxidized and Unoxidized (Controls) Wine Models

Total acetaldehyde was determined by HPLC with ultraviolet (UV) detection after previous derivatization with DNPH, as described by Han et al.^26^

Total SAs were analyzed by GC–MS analysis after derivatization with PFBHA. Briefly, samples are introduced within the anoxic chamber and 12 mL aliquots spiked with the internal standards (2-methylpentanal, 3-methylpentanal, phenylacetaldehyde-d2, and methional-d2). Samples are taken out and incubated at 50 °C for 6 h to ensure equilibration. After this, 360 μL of a 10 g/L PFBHA solution are added and the reaction is developed at 35 °C for 12 h 10 mL of the sample is then extracted in 1 mL cartridges packed with 30 mg of LiChrolut-EN resins. The cartridge is washed with 10 mL of a solution containing 60% methanol and 1% NaHCO_3_ and then dried and eluted with 1.2 mL of hexane. Three microliters of this extract are injected in the splitless mode in the GC–MS system.

Free PFMs are determined by GC–MS in the negative chemical ionization mode using the procedure described by Mateo-Vivaracho et al.^27^ Total PFMs are the sum of the free forms and those forming disulfides with themselves or with other mercaptans. For the determination of this total fraction, tris (2-carboxyethyl) phosphine is added to the sample in the anoxia chamber at a concentration of 1 mM prior to the analysis in order to reduce the disulfides back to mercaptans.^28^

Varietal aroma compounds, linalool, geraniol, and 1,1,6-trimethyl-1,2-dihydronaphthalene (TDN), are determined by GC–MS using the procedure described by López et al.^29^

Color was determined by the measurement of absorbances at 420, 520, and 620 nm as recommended by the OIV^30^ and total polyphenol index (TPI) by measurement at 280 nm.^30^

Tannin activity was measured as is described in the Supporting Information.

Redox potential was measured within the anoxic chamber with a commercial platinum electrode versus an Ag–AgCl(s) reference electrode (HI3148 HANNA, instruments, USA) in a potentiometer HI98191 also from HANNA.

### Data Analysis

Basic statistical analyses were carried out with Excel spreadsheet. Analysis of variance (ANOVA) was carried out with XLSTAT version 2015 (Addinsoft, XX). PLS modeling was carried out with Unscramble vs (Camo, Norway).

As main data were differences between oxidized samples and controls, their uncertainty was estimated by applying the basic theory of error propagation attending to the formula
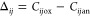

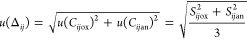
where *C*_*ij*ox_ and *S*_*ij*ox_ are the mean concentration and standard deviation of the three replicates of compound *j* in the oxidized sample *i*. *C*_*ij*an_ and *S*_*ij*an_ are the mean concentration and standard deviation of the three replicates of compound *j* in the anoxic control of sample *i*.

## Results and Discussion

The experimental setup is based on the preparation of wine models with standardized composition in metals, amino acids, PFMs, alcoholic degree, and pH, so that the single difference between the wine models in the study are the polyphenolic profiles extracted from the grapes. These were from different grape cultivars and different winemaking areas of Spain. The final reconstituted wine models were subjected to an oxidative aging treatment, in which samples were given 50 mg L^–1^ oxygen and were left for 35 days at 35 °C and to an equivalent storage in strict anoxia used as a control.

### Overview of Changes Introduced by Oxidation and Effect of Cultivar

**Figure 1:**
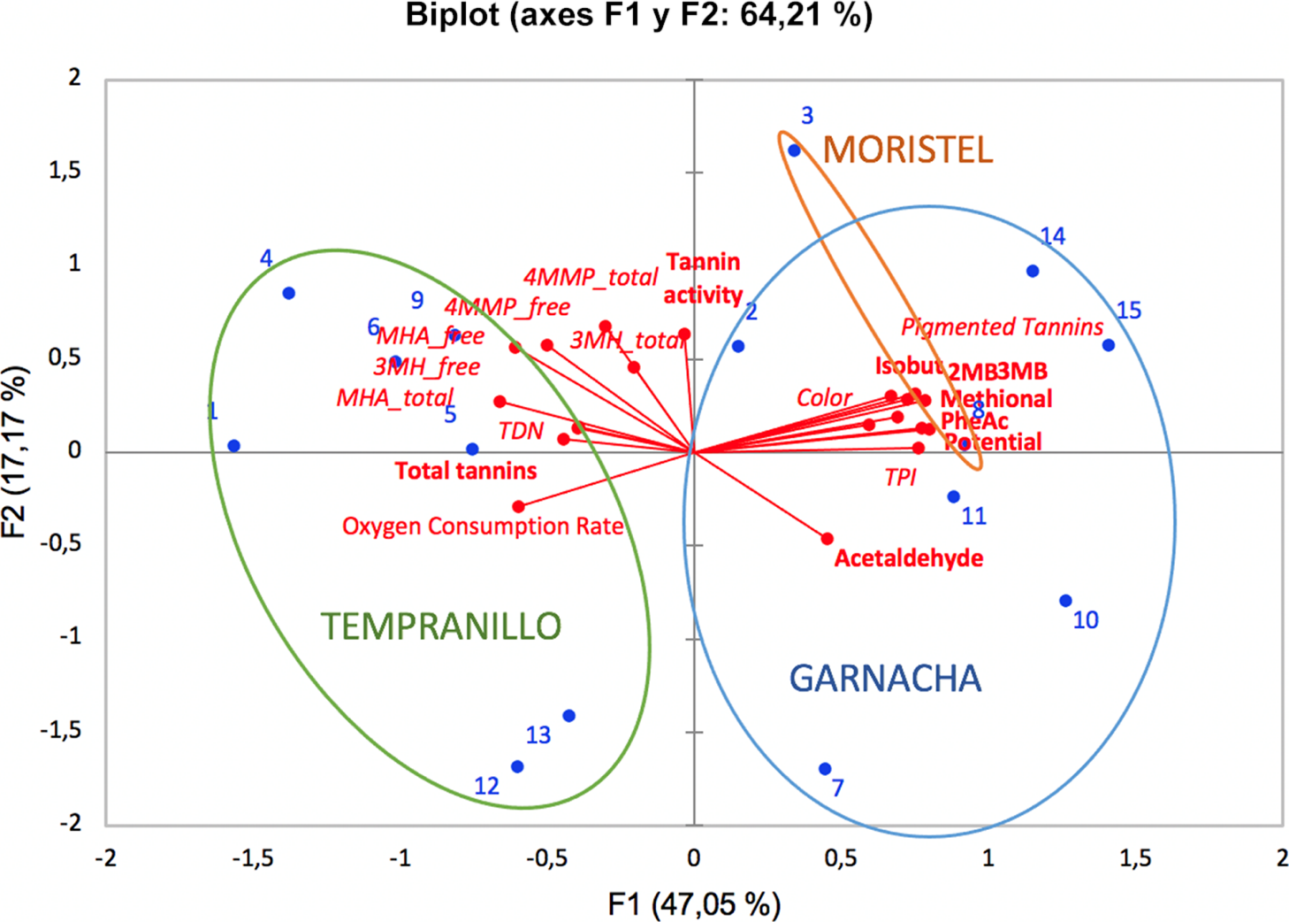
Overview of the differential response to oxidation induced by the polyphenolic profile. Projection of samples and variables in the plane of the two first dimensions of the PCA carried out on the data matrix containing OCRs and increases or decreases caused by oxidation vs the corresponding anoxic controls.

The major changes introduced by oxidation, in comparison with the corresponding anoxic controls, are summarized in Table 1 and in Figure 1 (the complete set of results of the experiment can be found in Supporting Information, Tables S1–S6). Data in Table 1 are the average increments (positive) or decreases (negative) caused by oxidation in the different compositional parameters registered for the individual samples (left part of the table) or averaged by cultivar (right part of the table).

**Table 1:** OCRs and Average Compositional Changes Introduced by Oxidation^a^^,^^b^

	average increments by sample				average increments by cultivar			
	max	min	average	*p*(F)	garnacha	tempranillo	moristel	*p*(F)
OCR (mg L^–1^ per day)	13.63	5.63	8.63	**6.95 10**^**–12**^	6.6	11.0	6.1	**1.06 10**^**–19**^
potential (mV)	214.73	64.03	133.7	**1.82 10**^**–27**^	167.6 a	84.28 b	204.65 a	**6.85 10**^**–06**^
color (CI)	0.71	–3.12	0.08	**1.56 10**^**–19**^	0.02 a	–1.42 ab	0.12 b	**0.018**
tannin activity (stickiness)	2065.8	–342.73	1058.8	**3.53 10**^**–22**^	1063.8 a	1101.64 a	894.28 a	0.89
IPT	0.27	–5.37	–1.84	**2.02 10**^**–11**^	–0.73 b	–3.31 c	0.02 a	**0.00014**
total tannins (mg L^–1^)	244.16	–153.51	51.59	**5.31 10**^**–18**^	16.53 a	69.27 a	94.9 a	0.54
pigmented tannins (mg L^–1^)	–17.15	–141.44	–81.84	**4.82 10**^**–10**^	–51.49 a	–113.69 b	–61.41 a	**0.00061**
acetaldehyde (mg L^–1^)	14.41	10.22	12.58	**0.00017**	13.07 a	12.24 a	12.33 a	0.68
isobutyraldehyde (μg L^–1^)	48.65	20.34	29.57	**4.10 10**^**–09**^	35.8 a	23.99 b	30.4 a	**0.025**
2-methylbutanal (μg L^–1^)	56.95	23.78	35.77	**3.24 10**^**–13**^	43.04 a	29.15 b	37.1 a	**0.009**
3-methylbutanal (μg L^–1^)	77.22	29.24	47.96	**6.47 10**^**–15**^	57.52 a	37.27 b	56.69 a	**0.0029**
methional (μg L^–1^)	178.72	69.85	126.11	**1.15 10**^**–24**^	148.81 a	97.22 b	159.17 a	**0.00023**
phenylacetaldehyde (μg L^–1^)	187.39	80.43	147.06	**2.81 10**^**–15**^	169.53 b	118.13 c	180.89 a	**0.00012**
linalool (a.u.)	1498.14	–4778.43	–250.84	**8.90 10**^**–10**^	–567.05 a	–72.93 a	75.14 a	0.71
geraniol (a.u.)	3973.90	–14559.79	140.69	**0.000028**	–1011.9 a	825.12 a	1202.68 a	0.7
TDN (a.u.)	100.07	–2395.42	–656.34	**1.85 10**^**–06**^	–1193.48 b	–265.36 a	–413.36 a	**0.04**
4MMP_free (μg L^–1^)	–34.72	–92.08	–64.8	**0.0198**	–76.45 b	–58.29 a	–52.68 a	0.083
MHA_free (μg L^–1^)	–9.51	–23.00	–17.17	**3.46 10**^**–07**^	–19.82 b	–14.63 a	–18.12 b	0.18
3MH_free (μg L^–1^)	–37.16	–92.22	–66.38	**1.53 10**^**–08**^	–77.11 b	–54.27 a	–76.6 b	**0.011**
4MMP_total (μg L^–1^)	–40.59	–106.28	–75.61	0.055	–87.16 b	–74.15 b	–46.10 a	**0.0027**
MHA_total (μg L^–1^)	–9.62	–38.15	–24.18	**1.73 10**^**–13**^	–26.34 b	–19.21 a	–35.07 c	**0.0018**
3MH_total (μg L^–1^)	–39.25	–100.5	–69.68	**0.049**	–79.74 a	–63.88 a	–59.81 a	0.09

a Except OCRs, data are the differences between oxidized samples and the corresponding anoxic controls. The left part of the table refers to differences, averaged by replicates, within samples; the right part to differences averaged by sample and cultivar. The significances of the differences are given by the corresponding *p*(F) obtained by ANOVA. Positive and negative values indicate increase or decrease (gray letters) with oxidation, respectively.

b a.u. Area units.

In general, the table reveals that oxidation causes increases of great magnitude in redox potential, tannin activity, and in the levels of SAs and increases of moderate magnitude in total tannins and acetaldehyde. Similarly, oxidation causes decreases of great magnitude in free and total PFMs and of moderate magnitude in TPI, pigmented tannins, and in TDN. Most of these changes were expected, although there are very few previous reports about tannin activity, and the decrease of TDN with oxidation has not been previously observed. Average levels of linalool and geraniol did not change significantly with oxidation.

As samples exclusively differ in their polyphenolic composition, differences between samples should be entirely attributed to differences in their specific or varietal polyphenolic profiles. The significance of the effects exerted by these profiles is assessed by means of the *p*(F) values obtained in the corresponding ANOVAs. Regarding specific sample effects, results in Table 1 reveal that the polyphenolic composition exerted a deep effect on the magnitude and in some cases even on the nature of the effects introduced by oxidation. In fact, changes in all measured chemical parameters, except in the total levels of 4MMP, were significantly related to the polyphenolic profile. Many of the changes were also significantly related to the grape cultivar, as can be seen in the last column of the table. Remarkably, increases in total tannins, acetaldehyde, and in tannin activity were not related to the cultivar.

The effects of the varietal polyphenolic profile are most clearly seen in the principal component analysis (PCA) plot given in Figure 1. The figure shows the projection of samples and variables in the plane of the two first principal components obtained from the data matrix containing oxygen consumption rates (OCRs) and the mean (average by replicates) increases or decreases caused by oxidation (vs the anoxic controls) in the 15 different samples. Note that in such figure, the directions of the variable loadings indicate higher increases for variables increasing with oxidation, but smaller decreases for those decreasing. In any case, the figure reveals the existence of a strong varietal influence because the samples containing polyphenols extracted from Tempranillo are clearly separated from those extracted from Garnacha and Moristel. Those containing polyphenols from Tempranillo consumed oxygen much faster, ended up with less residual oxygen and hence lower redox potential, lost more TPI, more pigmented tannins, and more color, but they lost less PFMs due to oxidation and accumulated smaller levels of SAs. Results will be commented and discussed in more detail later.

### OCRs and Redox Potential

OCRs were clearly varietal dependent, as can be seen in Table 1. Samples containing polyphenols from Tempranillo consumed in average 11.0 mg/L O_2_ per day in the first period of oxidation (4 days), while those from Garnacha consumed just 6.6 and those from Moristel 6.1 mg/L per day. The oxidation experiment was finished after 35 days, regardless of whether the O_2_ had been completely consumed or not. This means that samples consuming O_2_ more slowly contained higher final residual levels of O_2_, and consequently, higher redox potentials. Samples with PAFs from Moristel were particularly poor at O_2_ consumption, so that in the 35 days, they left unconsumed a total of 7.08 ± 2.2 mg of oxygen per liter of wine (accounting that remaining in the headspace) and their average redox potential was 190 mV. Those samples with PAFs from Garnacha left unconsumed just 2.87 ± 1.61 mg/L and ended with an average redox potential of 152 mV, while those from Tempranillo left just 1.24 ± 0.25 mg/L and ended with a redox potential of 60.5 mV.

OCRs were positively and significantly correlated to total tannins, to their mDP, to total prodelphinidins, and to the sample content in 3-monoglucoside anthocyanins (delphinidin, petunidin, and cyanidin), as summarized in Table 2. These correlations were expected. Delphinidin and prodelphinidins are easily oxidizable wine polyphenols due to the three vicinal hydroxy groups in the B ring^31^ and have been previously found correlated to OCRs. Anthocyanins are more reactive toward superoxide radical than catechin,^32^ and it is known that polymeric tannins are more antioxidant than monomeric forms.^33^

**Table 2:** Relationships between Some of the Changes Introduced by Oxidation or by the Anoxic Storage and the Initial Polyphenolic Composition of the Fractions Extracted from Grapes of Garnacha and Tempranillo^a^^,^^b^

		aldehyde accumulated						PFM remaining		
polyphenol or polyphenolic compositional parameter	OCR (1st 4 days)	acetaldehyde	isobutyraldehyde	2-methylbutanal	3-methylbutanal	methional	phenylacetaldehyde	4MMP free	MHA free	3MH free
mDP	0.78**									
total tannins	0.78**									
non pigmented tannins (mg L^–1^)	0.77**							–0.52#	–0.49#	–0.63*
non pigmented tannins (%)			0.68*	0.71**	0.72**	0.8**	0.73**			
prodelphinidins	0.83**		–0.62*	–0.68**	–0.72**	–0.84***	–0.84***			
sum of delphinidin, cyanidin and petunidin	0.59*		–0.62*	–0.64*	–0.66*	–0.7**	–0.62*			
delphinidin	0.63*									
color (CI)	0.65*		–0.72**	–0.77**	–0.79**	–0.87***	–0.85***			
flavanols	–0.74**		0.76**	0.81***	0.83***	0.9***	0.87***			
catechin	–0.77**		0.77**	0.82***	0.83***	0.89***	0.85***			
phenolic acids		0.62**	0.73**	0.79**	0.75**	0.81***	0.81***			
sum of galocatechin and epigalocatechin								0.63*	0.62*	0.74**
initial tannin activity		–0.59*						–0.52#	–0.65*	–0.59*

a Data are correlation coefficients between wine OCRs, levels of aldehydes accumulated during oxidation, levels of free PFMs remaining in anoxic controls and the initial compositional parameters. (#*p* = 0.1–0.05; *p = 0.05–0.01; **p = 0.01–0.001; ***p < 0.001).

b mDP: Mean degree of polymerization.

The negative correlations of OCRs with catechin and to the total content in flavanols, shown in Table 2 may be just statistical artifacts because in the present case, samples with higher levels of catechin and flavanols have also a lower concentration of anthocyanins.

### Color and Tannin Activity

Differences in color index introduced by oxygen were not very intense but follow a varietal pattern, as can be seen in Table 1. In the case of samples containing polyphenols from Garnacha and Moristel, the color remained mostly unchanged, while those extracted from Tempranillo lost in average 1.5 units of color, which represents a loss of 10% of the total color of the sample. This is related to their highest OCRs previously seen, confirming that anthocyanins are quickly oxidized.

Tannin activity refers to the specific enthalpy of interaction between tannins and a hydrophobic surface (polystyrene divinylbenzene HPLC column). This parameter has been related to the perception of astringency and dryness in mouth,^34^ and as seen in Table 1, it strongly and significantly increases with oxidation in most samples in a nonvarietal related way. Changes were not related to any polyphenolic compositional parameter. However, a significant positive correlation with the redox potential measured in the samples stored in anoxia was observed (leaving out one sample of Tempranillo, *r* = 0.71, significant at *p* = 0.0027). Although the true meaning of the redox potential in wine and wine-like media is controversial,^35^ in the complete absence of oxygen and in standardized model wine, it can be hypothesized that more negative values of redox potential should be related to higher levels of H_2_S and of mercaptans, including cysteine and GSH.^36^ As the single source of these compounds in our samples is the initial dosage, which was the same for all samples, differences should be most likely related to the specific reactivity of the polyphenolic fractions to mercaptans, as it will later be commented in the PFM section. Therefore, it can be hypothesized that stronger increases in tannin activity during oxidation may be linked to polyphenolic fractions most reactive to mercaptans.

### Accumulation of Acetaldehyde

During oxidative aging, less than expected amounts of acetaldehyde were accumulated. Differences between samples were significant but of low magnitude because levels accumulated ranged between 11 and 15 mg/L and were not related to the grape cultivar.

It should be noted that levels of acetaldehyde accumulated are very low, considering the large dose of O_2_ consumed and the absence of SO_2_. It can be estimated that if all 1-HER formed was transformed into acetaldehyde, levels formed were between 67.5 and 56.86 mg L^–1^, so that acetaldehyde accumulated is just 16–26% of the maximum expected. This mismatch should be attributed to the known ability of wine polyphenols to react with acetaldehyde^10,37^ and to the existence of antioxidants able to quench the 1-HER radical.^4,6^ At present it is not possible to assess the relative importance of these two processes in preventing acetaldehyde accumulation. Remarkably, the amount of acetaldehyde accumulated is positively correlated with the total amount of phenolic acids (see Table 2), which may suggest that the demonstrated 1-HER radical quenching ability of these compounds^4^ is not critical in determining acetaldehyde formation.

### Accumulation of SAs

The accumulation of SA is significantly related to the cultivar of grape from which polyphenols were extracted, as was clearly seen in Table 1 and in Figure 1. Samples containing polyphenols extracted from Tempranillo accumulated the smallest levels of these compounds, in average a 30% less, than samples containing polyphenols extracted from Garnacha or Moristel. Differences between samples were of notable magnitude and reached factors between 2.4 and 2.9. The two aldehydes reaching highest levels were methional and phenylacetaldehyde, which in one sample from Garnacha were found at 196 and 208 μg/L (1.88 and 1.73 μMol), respectively. Maxima levels reached by isobutanal, 3-methylbutanal, and 2-methylbutanal were 51, 57, and 86 μg/L (0.71, 0.66, and 1.00 μMol), respectively. Considering that amino acids were present in all the samples at the same concentration (10 mg/L, 60–85 μMol), this implies that methionine and phenylalanine are far more reactive than valine, leucine, and isoleucine, in agreement with previous observations.^16,38^

As summarized in Table 2, the accumulation of SA is positively and significantly correlated to the content on phenolic acids, monomeric flavanols, and nonpigmented tannins and negatively correlated to the contents in prodelphinidins, anthocyanins, and color. PLS models relating levels of SA accumulated in oxidation to the original chemical composition of the polyphenolic extracts, classified by structural families, are given in Table 3. The models built are quite satisfactory from the statistical point of view, being able to explain more than 77% of the original variance by cross validation in all cases. PLS models are quite simple and explain SA accumulation with just five general compositional variables: anthocyanins, phenolic acids, flavonols, flavanols, and mDP. Thus, the molecular conformation of these polyphenols, their functional groups and their level of polymerization are characteristics of the polyphenolic profile that define the ability of the samples to accumulate SA. Models are essentially equivalent for the 5 SAs because they are strongly correlated. Models would confirm what univariate correlation coefficients suggested: anthocyanins, flavonols, and more condensed tannins, all of which are relatively strong antioxidants, impede the accumulation of SA, while phenolic acids and flavanols, which in general are weaker antioxidants and could also be classified as more prone to form stable o-quinones, can react with amino acids by Strecker degradation, thereby promoting SA formation.

**Table 3:** PLS Models Relating the Observed Accumulation of SAs to the Polyphenolic Composition of the Samples^a^

	isobutyraldehyde	2-methylbutanal	3-methylbutanal	methional	phenylacetaldehyde
PCs	4	3	3	2	1
*R*2	0.957	0.895	0.903	0.965	0.843
*R*2 cross-validation	0.879	0.775	0.815	0.931	0.772
RMSE	1.301	2.36	3.17	5.778	12.558
RMSE cross-validation	2.393	3.77	4.79	8.777	16.525
slope	0.957	0.895	0.903	0.964	0.844
slope cross validation	0.887	0.795	0.807	0.9	0.713
anthocyanins	–0.194	–3.207 × 10–2	–5.019 × 10–2	–6.629	–0.22
phenolic acids	0.627	0.667	0.506	10.045	0.278
flavonols	0.175	–1.623 × 10–2	–0.155	–8.834	–0.138
flavanols	0.123	0.269	0.347	10.159	0.306
mDP	–0.259	–0.113	–0.192	–6.473	–0.237

a mDP: Mean degree of polymerization.

The negative coefficients of anthocyanins in Tables 2 and 3 were already observed in a previous report^16^ where they were tentatively attributed to the known ability of anthocyanins to react with aldehydes. However, these negative coefficients should be also related to the complex molecular rearrangements suffered by these molecules during oxidation. Several authors report that the quinone in the B ring formed in the oxidation of di- and trihydroxy anthocyanins, which presumably is the α-dicarbonyl undergoing the Strecker degradation of amino acids, is just a transitory state which quickly reduces taking electrons from the cleavage of the C ring.^39−43^ Attending to this, anthocyanins may act as sacrificial antioxidants, avoiding the Strecker degradation.

The negative correlation of prodelphinidins observed in Table 2, may be related to the higher electrophilic character of their quinones, as reported by Mouls and Fulcrand^44^ and Imran et al.^45^ Prodelphinidins are majorly formed by trihydroxylated flavan-3-ols. Such higher electrophilic character would make these quinones undergo different reactions with different nucleophiles, which would decrease their availability for the Strecker degradation of amino acids.

The higher antioxidant character of flavonols is related to their double bond in the 2–3 carbons and to the carbonyl in the C ring.^46,47^ Such double bond is conjugated with those in the quinone formed in the B ring, which introduces a rather different reactivity.^41^ Additionally, some flavonols, such as quercetin, form quinones with geometrical structures different from those of ortho-quinones, which may hamper the induction of the Strecker degradation.^41^ Finally, in the case of mDP, apart from the already higher antioxidant character or more condensed tannins,^48^ it may be thought that steric hindrance could limit the efficiency of the quinones^41^ to induce Strecker degradation.

All this contrasts with the demonstrated ability of flavanols, phenolic acids, and nonpigmented tannins to form quinones, which would explain the positive correlations.

Remarkably, all these observations are, in general, consistent with those made by Carrascón et al.^49^ These authors found that the highest consumptions of phenylalanine and methionine during wine oxidation took place in wines with low anthocyanin/tannin ratios and in wines with high levels of catechin and low levels of EGCs. These wines were also poor SO_2_ consumers, which may suggest that SO_2_ can react more efficiently with the highly electrophilic quinones of three-hydroxyl flavanols or of anthocyanins than with the quinones of dihydroxyl flavanols.

Contrary to these observations, it has been recently demonstrated^15^ that in wine models at 80 °C, catechin and EC have a limited ability to produce phenylacetaldehyde by Strecker degradation of phenylalanine, at least in comparison with caffeic acid, gallic acid, and 3,4-dihydroxybenzoic acid. Other researchers, working at higher temperatures^14^ also found similar results regarding the limited ability of catechins to form phenylacetaldehyde in relationship with smaller *ortho*-diphenols or triphenols, such as catechol, methyl catechol, or pyrogallol. This should be attributed to the nucleophilic character of the A ring of catechins, not present in the simple mononuclear phenols. In any case, our results strongly suggest that catechin and EC are among the most active phenolic compounds promoting Strecker degradation in wines.

It should be observed that little amounts of the aldehydes, except for 2-methylbutanal, were also found in samples stored in complete anoxia. In most of the cases, levels were marginal, generally less than 5% of the amounts accumulated in the oxidation procedure. In the two wines accumulating maxima levels of phenylacetaldehyde, however, the levels formed in anoxia of this compound were close to 30 μg/L. The high repeatability observed strongly suggests that it was not a problem with the anoxic procedure. It should be rather thought that, in spite of the care with which the experiment was carried out, the complete wine models already contained some α-dicarbonyl able to produce the reaction.

### Losses of PFMs by Oxidation

**Figure 2:**
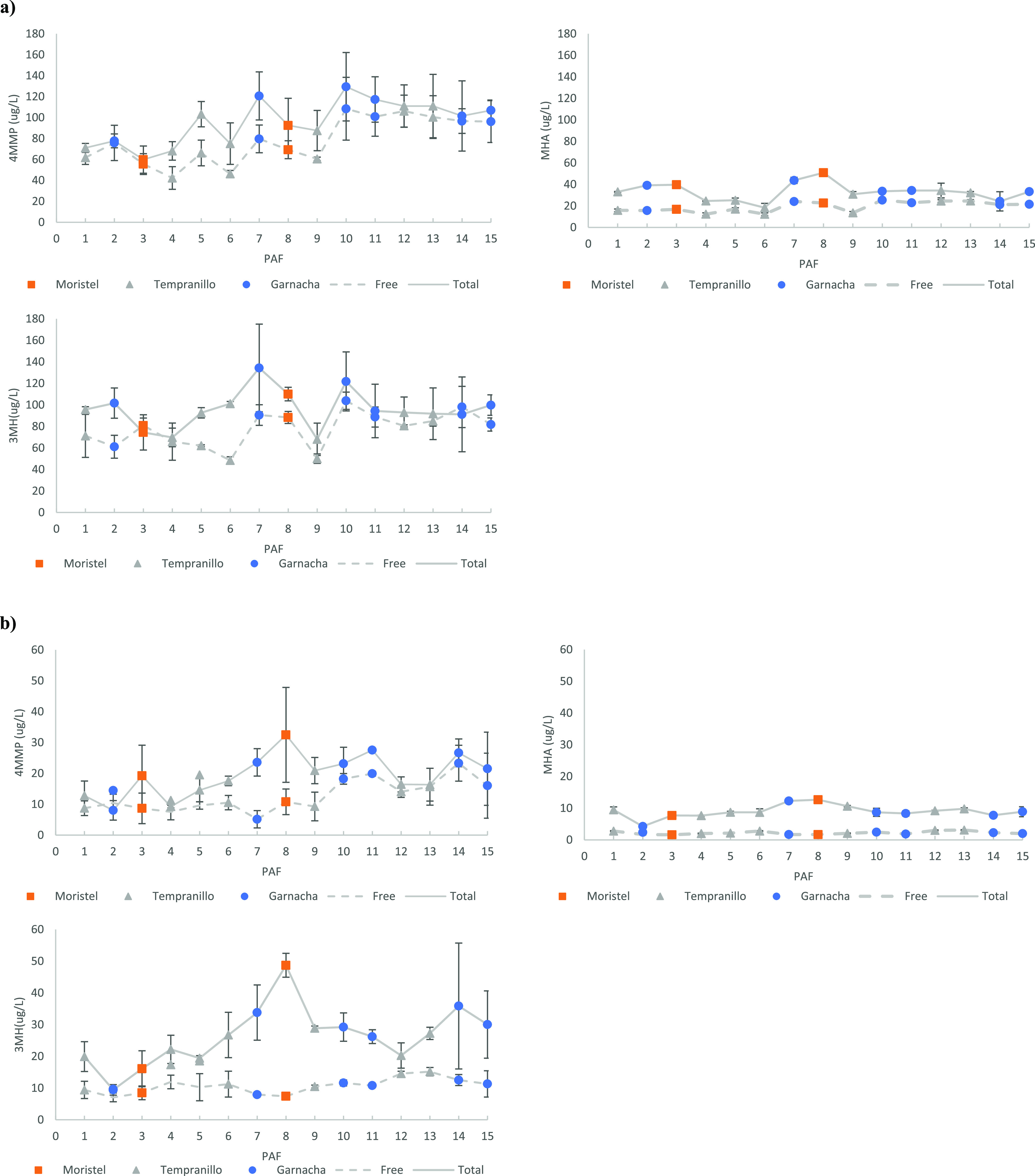
Levels of free (dotted lines) and total forms (solid lines) of polyfunctional mercaptans remaining after the experiment in anoxic controls (a) and in their corresponding oxidized samples (b). The error bars correspond to the deviation of the triplicates.

PFMs are key aroma components because they have a major role in the freshness and type of fruit perceived in wine aroma.^27^ Their presence is essential for wine longevity.^50^ In our experiment, the level of oxidation inflicted to the samples was very strong, so that levels of PFMs remaining after the oxidation were very low, as can be seen in Figure 2b. Free and total levels of 4MMP remaining were between 5 and 23 μg/L or between 8 and 27.5 μg/L, respectively; those of 3MH were between 7 and 15 μg/L and 9 and 48.7 μg/L, while free levels of MHA were in all cases smaller than 4.5 μg/L and total levels ranged between 4 and 13 μg/L. The comparison with the anoxic controls in Table 1 reveals that in the samples showing maximal decreases (column headed by min), more than 92% of free forms and 100% of the total forms of 4MMP and 3MH were lost by oxidation. Oxidative losses of MHA were comparatively smaller because this compound is also lost by chemical hydrolysis, yielding 3MH and acetic acid.

Perhaps because of such strong oxidation, differences between grape cultivars were not as clear. Only the decreases of free forms of 3MH and of total forms of 4MMP and MHA were significantly related to the cultivar (Table 1). There were, however, clear differences between samples and also between compounds, as can be seen in Table 1.

The difference between free and total forms should be attributed to the presence of disulfides likely formed with the GSH added to the models or alternatively to other mercaptans existing in the media, such as cysteine-rich native proteins coextracted with the polyphenolics, or to some mercapto-polyphenol formed during the preparation of the models. Disulfide formation may prevent the irreversible loss of PFMs by Michael addition on quinones. In fact, recent research has demonstrated that the stability of wines versus oxidation is strongly related to the presence of sulfur-containing compounds, mostly proteins.^51,52^ As levels of disulfides of the three analyzed PFMs are significantly correlated between them (*r* = 0.87, 0.79, and 0.69, significant at *p* < 0.0001, *p* = 0.0003, and *p* = 0.003, for the pairs 4MMP/MHA, 4MMP/3MH, and MHA/3MP, respectively), it seems that the ability to form disulfides is mostly a characteristic of each sample. Such a characteristic will be the result of the balance between the mercaptans present in the unoxidized sample and the number and activity of quinones formed during oxidation, as demonstrated by Nikolantonaki et al.^18,19^ Samples forming more reactive quinones and having less available mercaptans will hardly form disulfides. Remarkably, levels of disulfides of 4MMP and MHA were negatively correlated to the increase of tannin activity (significant at *p* = 0.046 and *p* = 0.004, respectively), which may suggest that the formation of those more active quinones is related to the increase of the tannin activity parameter.

Most remarkably, levels of total 4MMP surviving after the oxidation were significantly and negatively correlated to the sample content in pigmented tannins (*p* = 0.0073) and were also positively correlated with the decrease in pigmented tannins observed during the oxidation (*p* = 0.0029), which strongly suggests that 4MMP reacts during oxidation mainly with pigmented tannins. The reaction of grape tannins with volatile thiols has been described,^53^ but there are no previous reports about a special reactivity toward pigmented tannins. Such higher reactivity would be, however, compatible with their known more electrophilic character. In the case of MHA, surviving total levels after oxidation were negatively related to the increase observed in the tannin activity parameter. Remarkably, pigmented tannins are related to the formation of the sticky attribute.

PLS models in this case were not as successful as for SA (data not shown)

### Losses of PFMs under Anoxic Conditions

**Figure 3:**
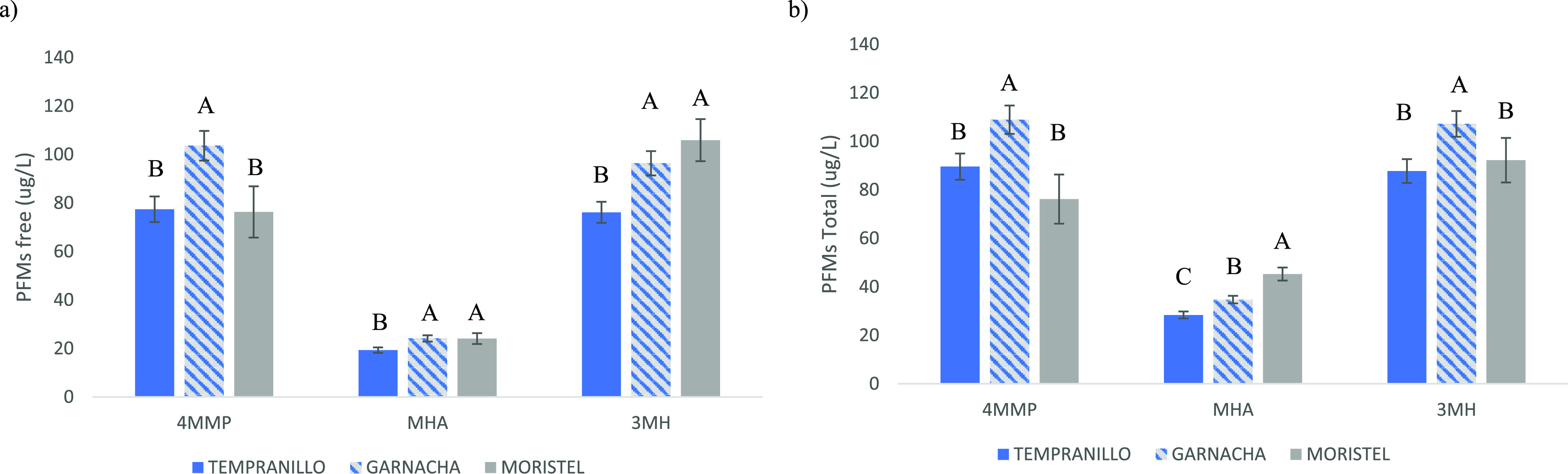
Levels of free (a) and total (b) PFMs remaining in the anoxic controls after the incubation period (initial levels were 100 μg/L). Different letters indicate significant differences between cultivars (Fischer posthoc test, *p* < 0.05). Error bars are standard errors of the means.

In the case of samples stored in anoxia, quite surprisingly, there were significant decreases in the free and in some cases also total levels of PFMs, as can be seen in Figure 2a and in Figure 3. Such decreases should be entirely attributed to the direct or indirect reactivity of the polyphenolic fraction toward PFMs and not to external oxidation processes because anoxia was strict. In some samples, such as sample 5, it is apparent that most of the decrease can be attributed to the reversible oxidation of the mercaptans to form disulfides. However, in some others, such as sample 3, the formation of disulfides was marginal at least for 4MMP and 3MH, so that in these samples, PFMs were irreversibly lost most likely by direct reaction with polyphenols. This reactivity was not expected. It should be considered that the PAFs were carefully extracted, and that once reconstituted, they were kept in an oxygen-free environment several weeks before the experiment in order to ensure that redox potential of the mixtures was negative. Results suggest, however, that such reductive potential may be not incompatible with the presence of some quinones presumably formed during sample preparation and responsible for the irreversible decrease of PFMs under anoxic conditions. On the other hand, unfermented grape extracted polyphenols should be more reactive than wine polyphenols toward mercaptans because wine polyphenols have been previously in contact with the little amounts of H_2_S and mercaptans produced by yeast during fermentation. More experimental work should be carried out to assess this.

In any case, there was a clear influence of grape cultivar on the levels of free and total forms remaining, as can be seen in Figure 3. The least reactive polyphenolic extracts were those of Garnacha, for which maxima levels were observed for the three PFMs. Remarkably, levels of PFMs remaining were positively correlated with the summation of the levels of GC and EGC and also with those of phenolic acids, and they were negatively correlated with nonpigmented tannins and with tannin activity, as can be seen in Table 2.

### Other Varietal Aroma Compounds

**Figure 4:**
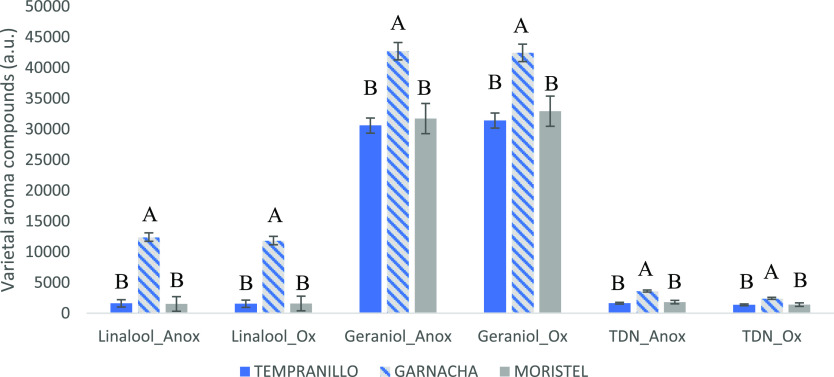
Levels of varietal aroma compounds remaining in the oxidation samples and anoxic controls after the incubation period. Different letters indicate significant differences between cultivars for each compound and condition (Fischer posthoc test, *p* < 0.05). Error bars are standard errors of the means.

In the present work, there was no fermentative aroma because all the work was carried out with unfermented samples. However, as aroma precursors were co-extracted with polyphenols, there was a significant development of some varietal aroma compounds during the anoxic or oxic storage of the samples. As in previous studies,^54^ some reports have suggested that oxidation may affect the aroma compounds, and we have specifically checked whether oxidation causes differences in the levels of at least three relevant varietal aroma compounds, such as linalool, geraniol, and TDN. Results are given in Figure 4 and reveal that even under the strong level of oxidation caused, there are no differences between levels of the selected varietal aroma compounds found in the anoxic controls and those found in the oxidized samples. This result contrasts with some previous observations by different authors, including ourselves, attending to which levels of linalool could be negatively related to the O_2_ consumed in the first saturation and not used to oxidize SO_2_ or to observed decreases of linalool of those samples stored at 50 °C under O_2_.^21^ In the latter study, there was also a clear increase in levels of TDN associated to the presence of O_2_. Results presented here demonstrate, however, that under normal storage conditions, levels of these aroma compounds are poorly affected by O_2_.

## Supporting Information Available

Chemical characterization of the PAFs; analytical conditions; cultivar, pH, acidity, brix grade, and geographical origin of the grape samples; polyphenolic indexes measured in the wine models after aging; OCRs measured in the experiment, redox potential, and acetaldehyde found in the wine models after the aging processes; levels of SAs found in the wine models after the aging processes; levels of varietal aroma compounds found in the wine models after the aging processes; levels of free polyfunctional mercaptans found in the wine models after the aging processes; and levels of free polyfunctional mercaptans found in the wine models after the aging processes (PDF)

## Abbreviations

1-HER: 1-hydroxyethyl radical
2MB: 2-methylbutanal
3MB: 3-methylbutanal
3MH: 3-mercaptohexanol
4MMP: 4-methyl-4mercaptopentanone
DNPH: 2,4-dinitrophenylhydrazine
CI: color index
EC: (-)-epicatechin
GC: (-)-gallocatechin
EGC: (-)-epigallocatechin
ECG: (-)-epicatechin gallate
GSH: glutathione
Ile: l-isoleucine
Isobut: isobutyraldehyde
Leu: l-leucine
mDP: mean degree of polymerization
MHA: 3-mercaptohexyl acetate
NCI: negative chemical ionization
PFM: polyfunctional mercaptan
PLS: partial least squares
Val: d-valine
OCR: oxygen consumption rate
PAF: polyphenolic aromatic fraction
PCA: principal component analysis
PFBHA: O-(2,3,4,5,6-pentafluorobenzyl)hydroxylamine hydrochloride
PG: polyethylene glycol
Phe: l-phenylalanine
PheAc: phenylacetaldehyde
SAs: Strecker aldehydes
TCEP: tris (2-carboxyethyl) phosphine
TDN: 1,1,6-trimethyl-1,2-dihydronaphthalene
TPI: total polyphenol index
UV: ultraviolet
