# Supplementary material for: Role of Grape-Extractable Polyphenols in the Generation of Strecker Aldehydes and in the Instability of Polyfunctional Mercaptans during Model Wine Oxidation
Source: J Agric Food Chem. 2021 Dec 13;69(50):15290–300. doi: 10.1021/acs.jafc.1c05880 (PMC8704169; doi:10.1021/acs.jafc.1c05880)
Supplement: Supplementary file 1 — jf1c05880_si_001.pdf [file jf1c05880_si_001.pdf]

The Role of Grape-Extractable Polyphenols in the Generation of Strecker Aldehydes and in the Instability of Polyfunctional Mercaptans during Model Wine Oxidation

Elena Bueno-Aventín[1], Ana Escudero[1], Purificación Fernández-Zurbano[2], Vicente Ferreira[1]

[1]Laboratorio de Análisis del Aroma y Enología (LAAE), Departamento de Química Analítica, Universidad de Zaragoza, Instituto Agroalimentario de Aragón (IA2) (UNIZAR-CITA), C/ Pedro Cerbuna 12, 50009 Zaragoza, Spain

[2] Instituto de Ciencias de la Vid y del Vino (Universidad de La Rioja, CSIC, Gobierno de La Rioja). Finca La Grajera, E-26007 Logroño, La Rioja, Spain

**Correspondence: vferre@unizar.es*

Supporting information

This material is available free of charge via the Internet at http://pubs.acs.org

I.- Chemical characterization of the PAFs: analytical conditions

Characterization of tannins

The analyses were carried out with an Ultra High Pressure Liquid Chromatography system (Shimadzu Nexera, KIOTO, JAPAN) coupled to a Photodiode Array Detector - SPD-M30A from Shimadzu (KIOTO, JAPAN). It was operated with Labsolutions software, using a PLRP-S 100 Å 3µm, 2.1 × 50 mm column (Agilent) protected with a PLRP-S 100Å 3µ × 5 mm guard column (Agilent). The samples were run at four column temperatures (30, 35, 40, and 45 °C). Tannin activity, also called stickiness, calculated as specific enthalpy of interaction between tannins and a hydrophobic surface (polystyrene divinylbenzene HPLC column), as proposed by Yacco et al., 2016 [1] was quantified. The concentration of total tannin and pigmented tannins were determined in chromatogram made at 30 ºC and they were reported in epicatechin equivalents and area data, respectively.

Mean degree of polymerization by phloroglucinol reaction

The protocol was made according to a previously described method of Arapitsas et al. (2). Briefly, a solution of 0.1 N HCl in MeOH, containing 100 g L-1 phloroglucinol and 20 g L^{-1} ascorbic acid was prepared. Then, 100 µL of the samples were reacted individually with 100 µL of the phloroglucinol solution at 50 °C for 30 min. In order to stop the reaction 1 mL of 40 mM aqueous sodium acetate was added. Samples were filtered by 0.22 µm before injection. Calibration curves were prepared with (+)-catechin, (−)-epicatechin (EC), (−)-gallocatechin (GC), (−)-epigallocatechin (EGC), (−)-epicatechin gallate (ECG), procyanidin B1, procyanidin B2, epicatechin 4-phloroglucinol, epicatechin-gallate 4-phloroglucinol and epigallocatechin 4-phloroglucinol for the quantification before the reaction and with the corresponding phloroglucinol adducts after reaction.

The quantification was carried out with an Acquity Ultra Performance Liquid Chromatographic system (Waters, MA, USA) coupled to a Xevo TQ MS System (Waters, UK) operating under MassLynx XS software, using a Waters Acquity HSS T3, 1.8 µm, 2.1 × 150 mm column (Waters), at 40 ºC. The compound detection was based on specific MS transitions in Multiple Reaction Monitoring (MRM) mode. The injection volume of 2 µL was employed for both the standard solutions and the samples. The chromatographic separation method used was that described by Arapitsas et al. [2].

UHPLC-MS/MS determination of anthocyanins

The samples were analyzed with an Acquity Ultra Performance Liquid Chromatographic system (Waters, MA, USA) coupled to a Xevo TQ MS System (Waters, UK) operating under MassLynx XS software. A reverse phase (RP) Acquity UPLC BEH C18, 1.7 µm, 2.1 × 150 mm column (Waters), protected with an Acquity UPLC BEH C18, 1.7 µm, 2.1 × 5 mm precolumn (Waters) was employed. The quantification was carried out using the method described by Arapitsas et al. [3].

UHPLC-MS/MS determination of polyphenols

Samples were analyzed with an Acquity Ultra Performance Liquid Chromatographic system (Waters, MA, USA) coupled to a Xevo TQ MS System (Waters, UK) operating

under MassLynx XS software. The chromatographic separation of the phenolic compounds was carried out in a Waters Acquity HSS T3 column, 1.8 µm, 2.1 × 150 mm (Waters). The method described by Vrhovsek et al. [4]was employed.

References

1. Yacco RS, Watrelot AA, Kennedy JA. Red Wine Tannin Structure-Activity Relationships during Fermentation and Maceration. J. Agric. Food Chem. **2016**;*64*(4):860–9.
2. Arapitsas P, Perenzoni D, Guella G, Mattivi F. Improving the Phloroglucinolysis Protocol and Characterization of Sagrantino Wines Proanthocyanidins. Molecules. **2021**;*26*(4):1–17.
3. Arapitsas P, Perenzoni D, Nicolini G, Mattivi F. Study of sangiovese wines pigment profile by UHPLC-MS/MS. J. Agric. Food Chem. **2012**;*60*(42):10461–71.
4. Vrhovsek U, Masuero D, Gasperotti M, Franceschi P, Caputi L, Viola R, et al. A Versatile Targeted Metabolomics Method for the Rapid Quantification of Multiple Classes of Phenolics in Fruits and Beverages. J. Agric. Food Chem. **2012**;*60*(36):8831–40.

II.- Supplementary data

Table S1: Cultivar, pH, acidity, brix grade and geographical origin of the grape samples.

Sample code	Cultivar	pH	Total acidity	ºBrix	Region of origin
1	Tempranillo	3.5	4.275	23.6	Somontano
2	Garnacha	3.23	4.875	26	Somontano
3	Moristel	3.31	5.16	24	Somontano
4	Tempranillo	3.61	4.05	25	La Rioja
5	Tempranillo	3.92	3.825	24.6	La Rioja
6	Tempranillo	3.37	5.5125	19.8	Ribera del Duero
7	Garnacha	3.41	4.3125	19	La Rioja
8	Moristel	3.38	5.421	23.5	Somontano
9	Tempranillo	3.52	4.237	25.1	Ribera del Duero
10	Garnacha	2.98	7.95	23.6	La Rioja
11	Garnacha	3.34	4.725	25.6	La Rioja
12	Tempranillo	3.5	4.35	24.6	Ribera del Duero
13	Tempranillo	3.63	5.475	25.2	Ribera del Duero
14	Garnacha	3.19	5.775	23	Somontano
15	Garnacha	3.41	5.962	27.8	La Rioja

Table S2: Polyphenolic indexes measured in the wine models after aging. Anoxic conditions are considered control treatments to assess effects of O_2.

	TPI		Tannin activity (Stickiness)		Total tannins (mgL^{-1})		Pigmented tannins (mgL^{-1})		Color (CI)	
PAF	Anoxic	Oxidation	Anoxic	Oxidation	Anoxic	Oxidation	Anoxic	Oxidation	Anoxic	Oxidation
1	43.3 ± 0.1	38.0 ± 1.9	2345 ± 60	3528 ± 33	2590 ± 69	2834 ± 11	801 ± 22	705 ± 5	18.7 ± 0.2	16.1 ± 0.0
2	28.6 ± 0.1	27.8 ± 0.2	3303 ± 94	4599 ± 59	1922 ± 49	2145 ± 19	461 ± 17	399 ± 9	8.6 ± 0.1	8.5 ± 0.0
3	20.4 ± 0.1	20.2 ± 0.1	1200 ± 58	2832 ± 58	1298 ± 6	1447 ± 4	347 ± 8	273 ± 6	6.9 ± 0.2	7.1 ± 0.0
4	37.7 ± 0.5	34.1 ± 0.3	2631 ± 58	3945 ± 98	2395 ± 15	2558 ± 8	698 ± 11	556 ± 14	12.5 ± 0.4	15.6 ± 0.0
5	35.6 ± 0.5	33.1 ± 0.5	3026 ± 113	4903 ± 292	2513 ± 21	2605 ± 115	585 ± 17	469 ± 32	12.0 ± 0.1	10.7 ± 0.0
6	35.5 ± 0.1	33.8 ± 1.2	3409 ± 39	4281 ± 260	2565 ± 19	2503 ± 49	630 ± 8	530 ± 25	11.5 ± 0.0	11.9 ± 0.1
7	28.2 ± 1.5	28.0 ± 0.7	3969 ± 88	3627 ± 246	1959 ± 24	2054 ± 47	484 ± 11	409 ± 14	10.5 ± 0.0	9.5 ± 0.0
8	20.4 ± 0.1	20.6 ± 0.1	2651 ± 27	2807 ± 72	1382 ± 27	1422 ± 8	321 ± 5	272 ± 5	6.4 ± 0.1	6.5 ± 0.0
9	32.2 ± 0.6	28.7 ± 2.2	2980 ± 33	4336 ± 121	2454 ± 20	2433 ± 8	579 ± 7	489 ± 10	10.2 ± 0.0	9.8 ± 0.0
10	18.7 ± 0.4	18.3 ± 0.2	3063 ± 167	3929 ± 33	1420 ± 9	1342 ± 11	299 ± 6	240 ± 8	5.7 ± 0.0	6.1 ± 0.0
11	27.3 ± 0.4	26.3 ± 0.1	3915 ± 32	5118 ± 123	2103 ± 9	1949 ± 10	370 ± 6	353 ± 42	7.3 ± 0.1	7.2 ± 0.0
12	41.0 ± 0.1	38.5 ± 0.4	2863 ± 26	3344 ± 129	2906 ± 37	2944 ± 43	840 ± 16	705 ± 16	17.1 ± 0.1	17.0 ± 0.1
13	36.1 ± 0.8	33.7 ± 0.1	2308 ± 60	2936 ± 175	2384 ± 6	2481 ± 30	738 ± 7	621 ± 11	16.2 ± 0.1	14.1 ± 0.0
14	26.1 ± 0.8	25.8 ± 0.8	3835 ± 277	6055 ± 300	1974 ± 68	2063 ± 61	376 ± 1	346 ± 17	6.9 ± 0.0	7.6 ± 0.0
15	24.7 ± 0.1	24.0 ± 0.3	2573 ± 24	3868 ± 295	1887 ± 3	1813 ± 15	397 ± 4	330 ± 4	7.4 ± 0.3	7.5 ± 0.0

Table S3: OCRs measured in the experiment, redox potential and acetaldehyde found in the wine models after the aging processes. Anoxic conditions are considered control treatments to assess effects of O_2.

	OCR (mgL^{-1} per day)	Potential (mV)		Acetaldehyde (mgL^{-1})	
PAF	Oxidation	Anoxic	Oxidation	Anoxic	Oxidation
1	8.98 ± 1.08	-20.5 ± 10.0	52.9 ± 6.1	1.38 ± 0.13	11.61 ± 0.94
2	7.93 ± 0.14	3.2 ± 6.7	122.1 ± 20.5	1.29 ± 0.03	12.56 ± 1.75
3	5.75 ± 1.48	3.1 ± 12.7	198.7 ± 1.4	2.60 ± 0.86	12.59 ± 0.34
4	8.97 ± 0.86	-17.6 ± 9.5	74.8 ± 4.5	1.60 ± 0.29	12.13 ± 0.17
5	13.30 ± 0.92	-47.1 ± 2.9	64.0 ± 4.3	1.29 ± 0.19	13.12 ± 1.27
6	11.33 ± 2.16	-14.6 ± 4.8	49.4 ± 12.8	1.53 ± 0.06	13.82 ± 3.34
7	5.63 ± 0.32	-38.0 ± 1.4	120.6 ± 7.7	1.45 ± 0.23	14.86 ± 1.85
8	6.47 ± 0.62	-31.3 ± 18.5	182.4 ± 2.8	1.00 ± 0.10	15.19 ± 0.14
9	9.23 ± 1.13	-10.4 ± 13.8	73.2 ± 8.5	1.43 ± 0.11	14.31 ± 0.82
10	6.00 ± 1.25	-27.8 ± 4.6	186.9 ± 5.7	1.16 ± 0.17	14.14 ± 0.97
11	8.33 ± 0.65	3.3 ± 17.6	150.3 ± 10.9	1.52 ± 0.55	14.70 ± 0.77
12	13.63 ± 1.33	-27.7 ± 5.6	50.6 ± 4.9	1.52 ± 0.12	15.04 ± 0.71
13	11.99 ± 1.04	-28.9 ± 3.0	58.3 ± 4.5	1.31 ± 0.20	15.72 ± 0.66
14	5.99 ± 0.33	-13.4 ± 6.0	164.7 ± 1.0	1.46 ± 0.10	14.66 ± 0.19
15	5.86 ± 0.19	-19.0 ± 15.9	169.3 ± 4.2	1.36 ± 0.15	15.74 ± 0.18

Table S4: Levels of Strecker aldehydes found in the wine models after the aging processes. Anoxic conditions are considered control treatments to assess effects of O_2.

	Isobutyraldehyde (ugL-1)		2-methylbutanal (ugL-1)		3-methylbutanal (ugL-1)		Methional (ugL-1)		Phenilacetaldehyde (ugL-1)	
PAF	Anoxic	Oxidation	Anoxic	Oxidation	Anoxic	Oxidation	Anoxic	Oxidation	Anoxic	Oxidation
1	0.70 ± 0.18	21.04 ± 2.76	0.00 ± 0.00	23.78 ± 2.69	1.06 ± 0.11	30.30 ± 3.39	1.87 ± 0.10	71.72 ± 1.85	3.77 ± 0.25	84.20 ± 3.72
2	0.89 ± 0.04	39.41 ± 8.16	0.00 ± 0.00	41.33 ± 6.56	1.80 ± 0.47	53.36 ± 10.28	2.07 ± 0.08	130.38 ± 11.15	6.94 ± 0.07	159.60 ± 11.49
3	3.12 ± 0.61	30.53 ± 4.70	0.00 ± 0.00	36.18 ± 3.73	3.44 ± 0.60	57.77 ± 4.49	3.54 ± 0.16	176.66 ± 3.03	28.73 ± 0.47	208.16 ± 1.99
4	0.70 ± 0.27	24.37 ± 1.89	0.00 ± 0.00	29.57 ± 1.08	1.06 ± 0.25	39.32 ± 2.00	2.01 ± 0.07	102.87 ± 8.09	4.68 ± 0.39	117.75 ± 11.28
5	1.12 ± 0.07	27.46 ± 4.36	0.00 ± 0.00	31.63 ± 5.33	1.61 ± 0.06	42.71 ± 7.14	2.47 ± 0.07	114.49 ± 14.77	7.57 ± 0.05	141.64 ± 19.34
6	0.78 ± 0.11	24.88 ± 5.85	0.00 ± 0.00	28.82 ± 5.64	1.27 ± 0.09	40.43 ± 7.34	1.83 ± 0.04	96.91 ± 16.19	4.61 ± 0.40	119.92 ± 22.35
7	0.51 ± 0.05	21.65 ± 1.33	0.00 ± 0.00	28.09 ± 0.93	1.97 ± 1.56	39.95 ± 0.87	1.87 ± 0.11	124.86 ± 3.33	5.59 ± 0.25	157.36 ± 3.22
8	1.43 ± 0.35	34.47 ± 1.78	0.00 ± 0.00	38.02 ± 1.90	1.40 ± 0.33	60.46 ± 2.65	1.65 ± 0.08	146.86 ± 3.33	6.01 ± 0.47	188.36 ± 5.47
9	1.29 ± 0.66	26.32 ± 4.44	0.00 ± 0.00	30.65 ± 4.83	2.32 ± 1.16	42.79 ± 7.30	4.64 ± 4.18	108.55 ± 12.97	9.79 ± 6.43	134.96 ± 16.67
10	0.62 ± 0.02	33.83 ± 0.87	0.00 ± 0.00	41.56 ± 1.16	1.13 ± 0.09	60.25 ± 1.81	1.75 ± 0.05	161.68 ± 9.22	8.28 ± 0.09	195.66 ± 9.42
11	1.14 ± 0.25	34.72 ± 3.44	0.00 ± 0.00	42.04 ± 2.80	2.72 ± 1.21	59.30 ± 5.36	1.97 ± 0.25	146.50 ± 7.09	6.95 ± 0.39	175.43 ± 6.48
12	0.89 ± 0.07	24.40 ± 1.53	0.00 ± 0.00	29.49 ± 1.95	1.40 ± 0.03	36.10 ± 2.82	2.61 ± 0.24	94.69 ± 3.46	5.76 ± 0.10	109.26 ± 5.11
13	0.66 ± 0.05	25.28 ± 2.26	0.00 ± 0.00	30.13 ± 2.24	1.19 ± 0.02	39.12 ± 3.54	2.33 ± 0.17	106.66 ± 5.77	6.21 ± 0.23	157.87 ± 37.71
14	0.87 ± 0.07	40.55 ± 5.97	0.00 ± 0.00	48.28 ± 5.87	3.39 ± 3.03	66.07 ± 5.06	6.07 ± 0.31	164.44 ± 0.56	7.98 ± 0.51	185.41 ± 1.32
15	2.10 ± 0.02	50.74 ± 9.67	0.00 ± 0.00	56.95 ± 8.38	4.47 ± 0.00	86.44 ± 10.93	4.53 ± 0.27	183.26 ± 3.92	29.45 ± 0.42	208.91 ± 2.81

Table S5: Levels of varietal aroma compounds found in the wine models after the aging processes. Anoxic conditions are considered control treatments to assess effects of O_2.

	Linalool (a.u.)		TDN (a.u.)		Geraniol (a.u.)	
PAF	Anoxic	Oxidation	Anoxic	Oxidation	Anoxic	Oxidation
1	1680 ± 202	1599 ± 148	2086 ± 223	2186 ± 500	27812 ± 2932	29466 ± 384
2	8634 ± 450	8936 ± 590	2994 ± 650	2552 ± 243	34000 ± 2544	36926 ± 3399
3	1868 ± 89	1977 ± 162	2191 ± 316	1742 ± 325	31868 ± 348	33817 ± 850
4	1728 ± 110	1552 ± 87	2099 ± 321	1716 ± 212	31169 ± 594	33298 ± 643
5	1748 ± 154	1749 ± 34	2178 ± 346	1806 ± 222	33591 ± 1273	29547 ± 4841
6	1903 ± 24	1800 ± 36	1143 ± 318	871 ± 23	29447 ± 5107	30120 ± 8156
7	7122 ± 209	6941 ± 263	5293 ± 183	3127 ± 272	38061 ± 2337	39791 ± 321
8	1164 ± 31	1206 ± 128	1442 ± 344	1065 ± 220	31569 ± 192	32026 ± 669
9	1242 ± 35	1158 ± 64	1609 ± 248	1111 ± 94	32454 ± 1122	32739 ± 595
10	11304 ± 553	11023 ± 444	2383 ± 628	1654 ± 115	38449 ± 3407	37039 ± 895
11	12225 ± 151	12263 ± 314	3542 ± 315	2481 ± 511	37921 ± 271	39190 ± 758
12	1670 ± 22	1525 ± 103	1161 ± 206	954 ± 109	30598 ± 401	31953 ± 748
13	1351 ± 34	1428 ± 101	1177 ± 203	950 ± 101	28912 ± 984	32636 ± 1514
14	15080 ± 1575	10302 ± 333	4974 ± 268	2578 ± 352	54315 ± 2056	39756 ± 1923
15	20103 ± 3148	21601 ± 1905	2526 ± 426	2158 ± 232	53390 ± 7737	61799 ± 5470

Table S6: Levels of free polyfunctional mercaptans found in the wine models after the aging processes. Anoxic conditions are considered control treatments to assess effects of O_2.

	4MMP free (ugL^{-1})		MHA free (ugL^{-1})		3MH free (ugL^{-1})	
PAF	Anoxic	Oxidation	Anoxic	Oxidation	Anoxic	Oxidation
1	61.75 ± 9.27	8.72 ± 2.38	15.92 ± 4.02	2.79 ± 0.80	71.17 ± 28.23	9.41 ± 2.72
2	75.77 ± 16.82	10.42 ± 2.84	15.68 ± 2.97	1.70 ± 0.22	61.07 ± 10.63	7.06 ± 1.37
3	55.57 ± 9.91	8.66 ± 4.95	16.84 ± 2.51	1.61 ± 0.29	80.80 ± 6.94	8.49 ± 2.17
4	42.26 ± 10.82	7.54 ± 3.65	12.34 ± 2.16	2.01 ± 0.04	65.81 ± 17.27	11.92 ± 3.04
5	66.17 ± 17.30	9.60 ± 1.20	17.04 ± 5.93	2.15 ± 0.32	62.06 ± 1.17	10.27 ± 4.27
6	46.18 ± 3.33	10.53 ± 2.32	12.30 ± 1.27	2.79 ± 0.69	48.40 ± 3.34	11.25 ± 4.08
7	79.61 ± 18.70	5.14 ± 2.80	24.11 ± 4.66	1.70 ± 0.29	90.53 ± 13.54	7.94 ± 0.67
8	69.25 ± 8.56	10.79 ± 4.15	22.71 ± 3.29	1.69 ± 0.23	88.32 ± 5.60	7.42 ± 0.89
9	60.50 ± 1.69	9.27 ± 6.56	13.57 ± 0.24	2.05 ± 0.21	50.03 ± 4.39	10.47 ± 0.64
10	108.41 ± 29.96	18.22 ± 1.72	25.44 ± 4.50	2.45 ± 0.23	103.84 ± 8.02	11.61 ± 0.94
11	100.80 ± 18.65	19.91 ± 1.06	22.97 ± 3.93	1.86 ± 0.10	88.70 ± 9.36	10.79 ± 0.63
12	106.14 ± 15.27	14.06 ± 2.59	24.54 ± 0.49	3.02 ± 0.01	80.50 ± 0.91	14.56 ± 1.06
13	100.42 ± 20.41	15.68 ± 5.97	24.61 ± 4.91	3.11 ± 0.23	84.95 ± 4.74	15.17 ± 1.31
14	96.60 ± 11.75	23.30 ± 5.81	21.22 ± 3.97	4.21 ± 0.51	98.06 ± 19.17	12.52 ± 1.73
15	96.14 ± 19.96	16.02 ± 10.53	21.51 ± 3.16	2.01 ± 0.64	81.73 ± 6.14	11.31 ± 4.15

Table S7: Levels of free polyfunctional mercaptans found in the wine models after the aging processes. Anoxic conditions are considered control treatments to assess effects of O_2.

	4MMP_Total (ugL^{-1})		MHA_Total (ugL^{-1})		3MH_Total (ugL^{-1})	
PAF	Anoxic	Oxidation	Anoxic	Oxidation	Anoxic	Oxidation
1	71.01 ± 6.09	12.77 ± 6.73	33.07 ± 0.52	9.49 ± 1.29	95.65 ± 3.76	19.93 ± 6.65
2	77.70 ± 9.37	8.02 ± 4.48	39.12 ± 3.22	4.26 ± 0.32	101.62 ± 19.87	9.43 ± 2.34
3	59.83 ± 13.01	19.24 ± 9.89	39.70 ± 3.39	7.71 ± 1.07	74.40 ± 16.37	16.10 ± 5.66
4	68.13 ± 8.85	9.24 ± 0.60	24.61 ± 0.44	7.66 ± 0.19	69.71 ± 8.65	22.19 ± 6.30
5	103.18 ± 12.08	14.57 ± 5.35	25.27 ± 2.03	8.71 ± 0.43	92.63 ± 4.79	19.43 ± 1.10
6	75.06 ± 19.82	17.56 ± 1.54	18.31 ± 4.06	8.69 ± 1.11	101.11 ± 2.06	26.73 ± 7.15
7	120.63 ± 22.96	23.56 ± 4.44	43.73 ± 2.85	12.28 ± 0.40	134.30 ± 40.75	33.80 ± 8.70
8	92.50 ± 25.86	32.47 ± 15.37	50.82 ± 2.55	12.67 ± 1.19	110.01 ± 6.29	48.70 ± 3.77
9	87.54 ± 19.25	20.91 ± 6.00	30.89 ± 2.44	10.63 ± 0.30	68.01 ± 15.02	28.86 ± 1.01
10	129.42 ± 32.66	23.15 ± 5.32	33.60 ± 0.72	8.71 ± 1.28	121.79 ± 27.44	29.23 ± 4.46
11	117.24 ± 21.71	27.54 ± 0.48	34.33 ± 0.75	8.34 ± 0.82	94.33 ± 24.85	26.21 ± 2.17
12	110.96 ± 20.23	16.43 ± 3.43	34.31 ± 6.82	9.19 ± 0.21	92.90 ± 14.40	20.26 ± 5.64
13	111.00 ± 30.23	16.34 ± 5.32	32.18 ± 1.17	9.80 ± 0.31	91.75 ± 24.02	27.24 ± 1.93
14	101.50 ± 33.53	26.65 ± 4.51	24.26 ± 8.94	7.76 ± 0.54	91.16 ± 34.75	35.86 ± 19.84
15	106.89 ± 9.81	21.50 ± 11.85	33.24 ± 1.13	8.88 ± 1.57	99.80 ± 9.52	30.02 ± 10.62
